# Trajectories in long-term condition accumulation and mortality in older adults: a group-based trajectory modelling approach using the English Longitudinal Study of Ageing

**DOI:** 10.1136/bmjopen-2023-074902

**Published:** 2024-07-11

**Authors:** Christos V Chalitsios, Cornelia Santoso, Yvonne Nartey, Nusrat Khan, Glenn Simpson, Nazrul Islam, Beth Stuart, Andrew Farmer, Hajira Dambha-Miller

**Affiliations:** 1 Primary Care Research Centre, University of Southampton, Southampton, UK; 2 Queen Mary University of London, London, UK; 3 Nuffield Department of Primary Care Health Sciences, University of Oxford, Oxford, UK

**Keywords:** epidemiology, geriatric medicine, public health

## Abstract

**Abstract:**

**Objectives:**

To classify older adults into clusters based on accumulating long-term conditions (LTC) as trajectories, characterise clusters and quantify their associations with all-cause mortality.

**Design:**

We conducted a longitudinal study using the English Longitudinal Study of Ageing over 9 years (n=15 091 aged 50 years and older). Group-based trajectory modelling was used to classify people into clusters based on accumulating LTC over time. Derived clusters were used to quantify the associations between trajectory memberships, sociodemographic characteristics and all-cause mortality by conducting regression models.

**Results:**

Five distinct clusters of accumulating LTC trajectories were identified and characterised as: ‘no LTC’ (18.57%), ‘single LTC’ (31.21%), ‘evolving multimorbidity’ (25.82%), ‘moderate multimorbidity’ (17.12%) and ‘high multimorbidity’ (7.27%). Increasing age was consistently associated with a larger number of LTCs. Ethnic minorities (adjusted OR=2.04; 95% CI 1.40 to 3.00) were associated with the ‘high multimorbidity’ cluster. Higher education and paid employment were associated with a lower likelihood of progression over time towards an increased number of LTCs. All the clusters had higher all-cause mortality than the ‘no LTC’ cluster.

**Conclusions:**

The development of multimorbidity in the number of conditions over time follows distinct trajectories. These are determined by non-modifiable (age, ethnicity) and modifiable factors (education and employment). Stratifying risk through clustering will enable practitioners to identify older adults with a higher likelihood of worsening LTC over time to tailor effective interventions to prevent mortality.

**STRENGTHS AND LIMITATIONS OF THIS STUDY**

- The main strength of this study is the use of a large dataset, the English Longitudinal Study of Ageing (ELSA), assessing longitudinal data to examine multiple long-term condition trajectories.
- The ELSA dataset is nationally representative of people aged 50 years and older, including a broad range of long-term conditions and sociodemographics.
- The measurement was limited to 10 long-term conditions, based on what was available in ELSA, which may not be exhaustive of all possible long-term conditions.
- The probability of being in a cluster membership is based on model assignment, which can lead to misclassification bias.

## Introduction

 Globally, the average life expectancy has risen from 66.8 years in 2000 to 73.4 years in 2019.^1^ By 2050, the population over 60 and 80 years will reach 2.1 billion and 426 million, respectively.^2 3^ This rise in longevity raises the risk of developing multimorbidity, which is the co-occurrence of two or more chronic diseases.^4^ The worldwide prevalence of multimorbidity among older people is reported to be between 55% and 98%,^5^ and in the UK, this is expected to rise from 54% in 2015 to 68% in 2035.^2^ Multimorbidity represents an ongoing challenge for healthcare systems because people with multimorbidity have worse care outcomes, including functional limitation and disability,^6 7^ higher service utilisation,^5^ mortality^8^ and poorer quality of life.^5^ Management of multimorbidity places considerable economic and logistical burdens on services traditionally organised around single disease models.^6^ There are a range of risk factors for multimorbidity, although these may vary ‘quantitively and qualitatively across life stages, ethnicities, sexes, socioeconomic groups and geographies’.^9^ The most significant risk factor in multimorbidity, in virtually all contexts, is older age.^9 10^ Other documented risk factors include low education, obesity, hypertension, depression and low physical function, which were generally positively associated with multimorbidity.^10^

While there is ample evidence of identified risk factors^7 9^ and adverse care outcomes for multimorbidity cross-sectionally to help understand the prevalence and patterns of long-term conditions (LTC), they provide little evidence on temporal elements, including patterns of LTC development over time.^8 10 11^ There is a paucity of longitudinal approaches examining patterns in the accumulation of diseases.^12^ Understanding the trajectory that an older adult will follow in the progression towards an increased number of LTCs could help predict when intervention is needed and inform targeted and earlier preventive interventions. To address this gap in the literature, this study aimed to classify older adults with LTC into clusters based on the accumulation of conditions as trajectories over time, characterise these clusters and quantify the association between derived clusters and all-cause mortality.

## Methods

### Data source and study population

The English Longitudinal Study of Ageing (ELSA) is a longitudinal cohort of people aged 50 years or older living in England.^13^ The ELSA cohort profile has been described in detail elsewhere.^14^ In summary, it included 12 099 people at study entry in 2002 with follow-up every 2 years with self-report questionnaires on physical and mental health, well-being, finances and attitudes around ageing over time. Four yearly additional nurse visits collected objective data such as anthropometric data.^13 15^ The ELSA is an open cohort, and refreshment samples were added depending on the proportional age requirement for ELSA, so the total number of people in this cohort was 15 091. Our baseline was wave 2 (2004/2005) of the ELSA cohort, the first collecting time point in the study of LTC with a 9-year follow-up to wave 6 (2012/2013), the most recent wave with available data on all-cause mortality status.

### Multimorbidity

Multimorbidity was defined as the presence of two or more of the following 10 conditions: hypertension, diabetes, cancer, lung disease, cardiovascular disease, stroke, mental health disorder, arthritis, Parkinson’s disease and dementia. These are self-reported by patients, relatives or carers and verified by nurse visits.^13^ These 10 conditions were available within the ELSA dataset based on our earlier work to define multimorbidity.^16 17^ After statistical consideration due to the small sample size and clinical discussion, we grouped some of the conditions as follows: people with depression were combined with mental health disorders, asthma was combined with lung disease, Alzheimer’s within dementia and, finally, those with heart attack, angina, heart murmur, abnormal heart rhythm and congestive heart failure combined into those with cardiovascular disease.

### All-cause mortality

All-cause mortality was reported by end-of-life interviews on waves 2, 3, 4 and 6 with relatives and friends after death.

### Covariates

Sociodemographic variables included were age, sex, ethnicity (defined as white/non-white), education, employment and marital status. The education variable was categorised into four groups: less than upper secondary level, upper secondary or vocational level, tertiary level and others. Employment status was categorised into ‘paid employment’ and ‘unemployed’. Marital status was categorised into three groups: never married, married/having a partner and separated/divorced/widowed. These covariates were based on the baseline. We used data provided in the nearest subsequent waves if they were missing at baseline.

### Statistical analysis

Descriptive statistics were used to summarise participants’ characteristics. We used group-based trajectory modelling (GBTM) to classify older adults with LTC into clusters based on accumulating conditions as trajectories over time. GBTM is a finite mixture model applying maximum likelihood to identify a cluster of people following similar trajectories by the number of conditions over time.^18^ This model assumes the same error variance for all clusters and time points and treats missing data as ‘missing at random’.^19^ The procedure for selecting the best model included two steps: identifying the ideal number of trajectory groups and determining polynomial orders to represent the shapes of the trajectories.^18 20^ Based on the observed distribution, we employed a censored normal model to specify LTC.^21 22^ We fitted the models iteratively, starting with one and increasing up to a maximum of six clusters that would be useful in a clinical setting.^20^ We selected the number of trajectory clusters based on the following criteria: the lowest Bayesian information criterion value, average posterior probability assignment >70%, odds of a correct classification >5 and the percentage of participants in each trajectory group >5% of the total sample (if less than 5% it is unlikely to be conceptually useful for clinical practice).^22^,^24^ We first used cubic polynomials to characterise the shape of the clusters of LTC trajectories. However, after selecting the number of trajectories, we refitted the model to use lower order terms when the higher order terms were insignificant.^20^ We then assigned individuals to the trajectory group based on the maximum posterior probability.^20^ Multinomial logistic regression was then performed to test the association between sociodemographic factors and clusters of LTC trajectory, with the ‘no LTC’ cluster as the reference. Binary logistic regression was also performed to quantify the association between the clusters of LTC trajectory membership and all-cause mortality, adjusting for all the covariates mentioned above. A squared term of age was included in the model to account for the non-linear relationship between age and mortality. The significance level was set at a p value <0.05, and all analyses were performed using STATA MP V.17.0.

### Patient and public involvement

This study was conducted as part of a wider mixed-methods programme of research exploring the potential of machine learning to address multimorbidity through the ‘clustering’ of patients based on similarities in clinical and social care needs. Patient and public involvement has been incorporated throughout the wider research programme from the initial inception, design and dissemination of findings. The initial results and the final written draft of the study submitted in this manuscript were shared with our programme’s patient and public representative.

## Results

### Participants’ characteristics

**Figure 1:**
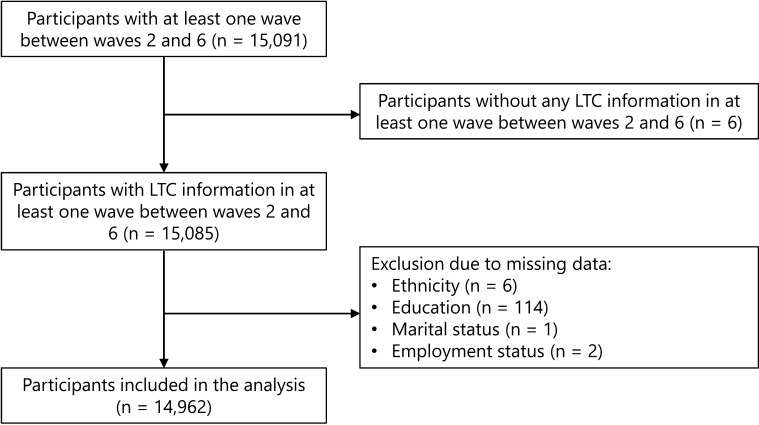
Flow chart of participant selection. LTC, long-term conditions; MLTC, multiple long-term conditions.

There were 9170 participants in wave 2 and we identified 15 091 individuals participating in at least one wave during the follow-up period. (The flow of participants through the study is shown in figure 1.) Six participants were excluded, as they had no information on LTC. After excluding those (n=123) with missing data on covariates, 14 962 people were included in the final analysis. The current analysis included 2688 (18.0%), 529 (3.5%), 4270 (28.5%), 4582 (30.6%) and 2893 (19.3%) people from waves 2, 3, 4, 5 and 6, respectively. The mean (SD) age of the cohort was 61.9^11^ years; most were females (53.5%), of white ethnicity (96.5%), with educational attainment of upper secondary or vocational level (43.1%), employed (56.8%) and married or had a partner (72%) (table 1).

**Table 1:** Participants’ characteristics overall and stratified by clusters of LTC trajectory

	Total14 962 (100%)	No LTC2826 (18.9%)	Single LTC4802 (32.1%)	Evolving multimorbidity3739 (25.0%)	Moderate multimorbidity2532 (16.9%)	High multimorbidity1063 (7.1%)
Age, mean (SD)	61.9 (11)	56.0 (9.1)	60.0 (10.0)	62.9 (10.8)	67.1 (10.7)	69.8 (10.4)
Sex						
Male	6951 (46.5)	1402 (20.2)	2361 (34.0)	1675 (24.1)	1050 (15.1)	463 (6.7)
Female	8011 (53.5)	1424 (17.8)	2441 (30.5)	2064 (25.8)	1482 (18.5)	600 (7.5)
Ethnicity						
White	14 440 (96.5)	2726 (18.9)	4629 (32.1)	3618 (25.1)	2451 (17.0)	1016 (7.0)
Non-white	522 (3.5)	100 (19.2)	173 (33.1)	121 (23.2)	81 (15.5)	47 (9.0)
Education						
Less than upper secondary	5107 (34.1)	629 (12.3)	1417 (27.8)	1326 (26.0)	1136 (22.2)	599 (11.7)
Upper secondary, vocational	6444 (43.1)	1399 (21.7)	2186 (33.9)	1609 (25.0)	941 (14.6)	309 (4.8)
Tertiary	2277 (15.2)	626 (27.5)	859 (37.7)	497 (21.8)	227 (10.0)	68 (3.0)
Others	1134 (7.6)	172 (15.2)	340 (30.0)	307 (27.1)	228 (20.1)	87 (7.7)
Employment						
Paid employment	8500 (56.8)	895 (10.5)	2278 (26.8)	2333 (27.5)	2033 (23.9)	961 (11.3)
Unemployed	6462 (43.2)	1931 (30.0)	2524 (39.1)	1406 (21.8)	499 (7.7)	102 (1.6)
Marital status						
Never married	789 (5.3)	148 (18.8)	268 (34.0)	189 (24.0)	131 (16.6)	53 (6.7)
Married/partner	10 766 (72.0)	2282 (21.2)	3635 (33.8)	2674 (24.8)	1566 (14.6)	609 (5.7)
Separated/divorced/widowed	3407 (22.8)	396 (11.6)	899 (26.4)	876 (25.7)	835 (24.5)	401 (11.8)

The percentages in the ‘Total’ column are presented vertically, whereas horizontally in the other five columns.

LTC, long-term condition.

### Clusters of LTC trajectory

We examined one to six clusters in the model to determine the optimal cluster number. Five clusters were selected using the model fit indicators (online supplemental table 1) and the interpretability of classified trajectories.^25^

**Figure 2:**
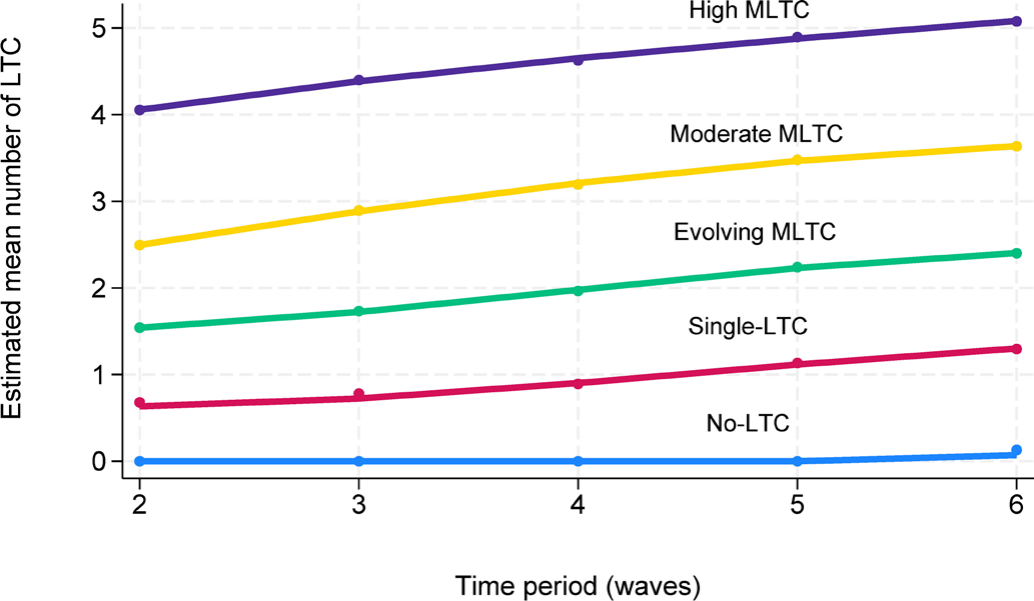
Clusters of long-term condition (LTC) trajectories over time (waves 2–6) in the English Longitudinal Study of Ageing study. The solid lines represent the estimated mean count of LTC profiles for the five clusters. The ‘no LTC’ cluster included people who did not have any of the examined LTC; the ‘single LTC’ cluster included those who did not develop multiple long-term conditions (MLTC) but may have had one LTC; the ‘evolving MLTC‘ cluster included those who developed MLTC lately; the ‘moderate MLTC’ cluster included those who started with the lower number of MLTCs and developed further LTC; the ‘high MLTC’ cluster consisted of those who started with the higher number of MLTCs and developed additional LTC.

Participants displayed high posterior probabilities of belonging to their assigned clusters ranging from 0.88 to 0.97 across the five clusters. The ‘no LTC’ cluster (18.57%) was dominated by people (95.2%) without any record of the examined LTC during the follow-up, and the ‘single LTC’ cluster (31.21%) consisted of those who did not develop multimorbidity during the study period but may have had one LTC (figure 2). The ‘evolving multimorbidity’ cluster (25.82%) was characterised by people who progressed from less than two LTCs at baseline to two, three or four by the end of follow-up. Two clusters had multimorbidity profiles which showed increasing numbers of LTCs (‘moderate multimorbidity’ (17.12%) and ‘high multimorbidity’ (7.27%)). Those in these clusters started with multimorbidity and continued to have higher counts of LTCs in the following periods.

### Clusters of LTC trajectory and sociodemographic characteristics

Increasing age was consistently associated with all LTC clusters, compared with the ‘no LTC’ cluster (tables1  2). Females had higher odds (adjusted OR (aOR)=1.13; 95% CI 1.01 to 1.27) of being in the ‘moderate multimorbidity’ clusters than males. Being non-white increased the odds of belonging to the ‘high multimorbidity’ cluster by 2.04 times (aOR=2.04; 95% CI 1.40 to 3) compared with being white. Higher education and paid employment decreased the odds of belonging to any of the four clusters than those with less than upper secondary education and unemployment, respectively.

**Table 2:** The association between sociodemographic factors and clusters of LTC trajectories

	Adjusted OR (95% CI) (Reference: no LTC)			
**Sociodemographics**	**Single LTC**	**Evolving multimorbidity**	**Moderate multimorbidity**	**High multimorbidity**
Age	1.04 (1.03 to 1.04)	1.05 (1.05 to 1.06)	1.07 (1.06 to 1.08)	1.08 (1.07 to 1.09)
Sex				
Male	Reference	Reference	Reference	Reference
Female	1.00 (0.91 to 1.10)	1.11 (0.99 to 1.23)	1.13 (1.01 to 1.27)	0.95 (0.81 to 1.11)
Ethnicity				
White	Reference	Reference	Reference	Reference
Non-white	1.17 (0.91 to 1.50)	1.13 (0.85 to 1.49)	1.36 (1.00 to 1.86)	2.04 (1.40 to 3.00)
Education				
Less than upper secondary	Reference	Reference	Reference	Reference
Upper secondary, vocational	0.92 (0.81 to 1.03)	0.87 (0.77 to 0.99)	0.77 (0.67 to 0.88)	0.53 (0.45 to 0.64)
Tertiary	0.84 (0.72 to 0.97)	0.68 (0.58 to 0.80)	0.51 (0.42 to 0.62)	0.33 (0.25 to 0.45)
Others	1.01 (0.83 to 1.25)	1.04 (0.84 to 1.28)	0.99 (0.79 to 1.25)	0.76 (0.57 to 1.02)
Employment				
Unemployed	Reference	Reference	Reference	Reference
Paid employment	0.79 (0.70 to 0.89)	0.54 (0.48 to 0.62)	0.35 (0.31 to 0.40)	0.17 (0.13 to 0.21)
Marital status				
Never married	Reference	Reference	Reference	Reference
Married/partner	0.85 (0.69 to 1.04)	0.90 (0.72 to 1.14)	0.80 (0.62 to 1.03)	0.82 (0.58 to 1.15)
Separated/divorced/widowed	0.97 (0.77 to 1.23)	1.14 (0.88 to 1.48)	1.27 (0.96 to 1.68)	1.41 (0.98 to 2.04)

LTC, long-term condition.

### Clusters of LTC trajectory and all-cause mortality

The ‘single LTC’ (aOR=1.81; 95% CI 1.21 to 2.73), the ‘evolving multimorbidity’ (aOR=2.26; 95% CI 1.51 to 3.38), the ‘moderate multimorbidity’ (aOR=2.62; 95% CI 1.75 to 3.94) and the ‘high multimorbidity’ (aOR=4.03; 95% CI 2.64 to 6315) clusters showed an association between increasing rates of all-cause mortality relative to the severity and complexity of multimorbidity (table 3).

**Table 3:** Association between clusters of LTC trajectory and all-cause mortality.

	Alive (14 310, 95.6%)	Dead (652, 4.4%)	Unadjusted OR (95% CI)	Adjusted* OR (95% CI)	P value†
Trajectory cluster					
No LTC	2796 (98.9)	30 (1.1)	Reference	Reference	<0.0001
Single LTC	4668 (97.2)	134 (2.8)	2.69 (1.81 to 4.01)	1.81 (1.21 to 2.73)	
Evolving multimorbidity	3566 (95.4)	174 (4.6)	4.59 (3.10 to 6.78)	2.26 (1.51 to 3.38)	
Moderate multimorbidity	2349 (92.8)	183 (7.2)	7.22 (4.89 to 10.7)	2.62 (1.75 to 3.94)	
High multimorbidity	931 (87.6)	132 (12.4)	13.6 (9.11 to 20.3)	4.03 (2.64 to 6.15)	

* Adjusted for age, sex, ethnicity, education, employment status and marital status. Age was included in the model as a squared term.

† P value for trend.

LTC, long-term condition.

## Discussion

This study examined clusters of LTC based on the accumulation of conditions as trajectories over time, their associations with sociodemographic factors and all-cause mortality among older adults in England. We identified five distinct clusters that can be described as ‘no LTC’, ‘single LTC’, ‘evolving multimorbidity’, ‘moderate multimorbidity’ and ‘high multimorbidity’. We observed that the accumulation of LTC over time progresses differently among older adults with distinction by ethnicity, educational level and employment status. Specifically, ethnic minorities showed faster/steeper progression towards increased numbers of LTCs, whereas higher education and paid employment had a protective effect on the increase in the accumulation of LTC.

Similar to an earlier study, we also found clusters that started with multimorbidity and continued to have higher counts of LTCs in the following periods, demonstrating individual variations in the progression of health decline.^25^ Other existing work has also shown variations in rates of LTC.^26^ No trajectories were identified demonstrating that health had improved over time (indicated by falling numbers of LTCs), a finding that aligns with the existing literature.^25 26^ This finding may indicate there is limited recovery from LTC in older adults or the result of an older population cohort where the mean number of conditions will likely increase over time (waves).^25^

The faster and steeper progression observed towards increased numbers of LTCs in females aligns with previous research, which found that older females accumulated morbidities at a faster rate than most other cohorts.^27^ An explanation could be that females tend to live longer than males, and as a result, they are more likely to develop chronic conditions associated with ageing, such as arthritis and dementia. The faster development of multiple long-term conditions (MLTC) in ethnic minorities can be explained by evidence, suggesting that access and engagement with healthcare are limited for some population groups, often on the basis of ethnicity. Specifically, a review from NHS Race and Health Observatory^28^ suggests that there are clear barriers for people from minority ethnic backgrounds to seek help for mental health problems, and another research has also found lower access to cancer screening in the UK.^29^ Socioeconomic risk factors are known to be associated with MLTC.^30^ Our findings support the role of higher educational attainment, a major socioeconomic risk factor, on MLTC prevention. Targeting education inequality is expected to lead further to the restriction of worsening MLTC. The effect of educational attainment on MLTC is thought to be explained by other risk factors that may mediate this association, such as body mass index and smoking.^31^

Over their life course, individuals develop MLTC. It is necessary to challenge the common statement that MLTC is inevitable in an ageing society. To do this, the focus on MLTC should shift from sole management of high-risk older individuals to include integrated population-level prevention strategies throughout the life course to address the drivers of MLTC. As Vetrano *et al* observe, knowledge of how LTCs cluster and how the health trajectories of individuals with multimorbidity change over time can increase understanding of the complexity and dynamic evolution of multimorbidity clusters, as well as supporting clinicians who manage co-occurring LTC and health policymakers who plan care resource use.^32^

This is the first study to examine trajectories of MLTC with a view to stratifying within MLTC to identify those at greatest risk among older adults in England. The main strength of the current study is the use of a large dataset, assessing longitudinal data to examine MLTC trajectories, and a dataset that is nationally representative of people aged 50 years and older, including a wide range of LTC and sociodemographics. However, this study has several limitations. First, the measurement of MLTC used was limited to the 10 LTCs available in the ELSA database, which only encompasses a relatively limited number of possible LTCs. Therefore, the results may have been different if more conditions were included in our analysis. Second, although we examined the correlates of MLTC trajectories using the variables measured at the baseline (wave 2), we cannot conclude on the directionality of the associations. Similar to other studies with a longitudinal design that have investigated age-related changes in multimorbidity over time, there is likely to be a confounding of age and period effects.^25^ Lastly, the probability of being in a cluster membership is based on model assignment, which can lead to misclassification bias.

To conclude, our work concurs with Vetrano *et al*’s observation that health trajectories of older adults with multimorbidity are typically characterised by dynamism and complexity but can still be tracked over time.^32^ Our findings contribute to existing evidence on the need to develop effective tailored interventions for at-risk individuals. Possible responses include targeting ethnic minorities for multimorbidity prevention. Additionally, higher levels of education can also lead to a further decrease in the number of LTCs. Policymakers should also commit to increasing MLTC awareness among at-risk groups and care providers.

## Supplementary material

10.1136/bmjopen-2023-074902 online supplemental file 1

## Data Availability

Data may be obtained from a third party and are not publicly available.
