# Supplementary material for: Trajectories in long-term condition accumulation and mortality in older adults: a group-based trajectory modelling approach using the English Longitudinal Study of Ageing
Source: BMJ Open. 2024 Jul 11;14(7):e074902. doi: 10.1136/bmjopen-2023-074902 (PMC11243147; doi:10.1136/bmjopen-2023-074902)
Supplement: online supplemental file 1 [file bmjopen-14-7-s001.pdf]

Trajectories in long-term conditions accumulation and mortality in older adults: A group-based trajectory modelling approach using the English Longitudinal Study of Ageing

Christos V. Chalitsios[1], Cornelia Santoso[1], Yvonne Nartey[1], Nusrat Khan[1,] Glenn Simpson[1], Nazrul Islam[1], Beth Stuart[2], Andrew Farmer[3], Hajira Dambha-Miller[1]

Supplements

Supplementary Table 1. Statistical parameters of the optimal number of clusters selection.

Number of groups	Group membership		Trajectory shapes	BIC (sample size=15085)	APPA	OCC
**1**	(1)	100	3	-85493.21	1	N/A
**2**	(1)	53.49	33	-73870.19	0.94	12.80
	(2)	46.51			0.94	17.71
**3**	(1)	21.77	333	-63524.35	0.97	105.25
	(2)	53.83			0.96	18.03
	(3)	24.40			0.95	67.92
**4**	(1)	19.24	3333	-59262.14	0.96	93.69
	(2)	36.07			0.93	24.44
	(3)	32			0.90	19.03
	(4)	12.69			0.96	172.34
**5**	(1)	19.35	33333	-56474.28	0.97	119.07
	(2)	30.77			0.90	18.95
	(3)	25.43			0.88	23.76
	(4)	17.15			0.90	44.02
	(5)	7.31			0.95	284.50
**6**	(1)	15.57	333333	-57000.83	0.96	109.69
	(2)	29.21			0.90	19.32
	(3)	23.82			0.87	20.91
	(4)	15.12			0.90	44.32
	(5)	6.27			0.95	259.29
	(6)	10.6			0.92	221.23

Note: Trajectory shapes (0=intercept, 1=linear, 2=quadratic, 3=cubic); BIC = Bayesian Information Criterion; APPA = average posterior probability assignment; OCC = odds of a correct classification according to maximum posterior probability group.
