# Supplementary material for: Trajectories in long-term condition accumulation and mortality in older adults: a group-based trajectory modelling approach using the English Longitudinal Study of Ageing
Source: BMJ Open. 2024 Jul 11;14(7):e074902. doi: 10.1136/bmjopen-2023-074902 (PMC11243147; doi:10.1136/bmjopen-2023-074902)
Supplement: Supplementary file 2 [file bmjopen-2023-074902.reviewer_comments.pdf]

PEER REVIEW HISTORY

BMJ Open publishes all reviews undertaken for accepted manuscripts. Reviewers are asked to complete a checklist review form (**http://bmjopen.bmj.com/site/about/resources/checklist.pdf**) and are provided with free text boxes to elaborate on their assessment. These free text comments are reproduced below.

ARTICLE DETAILS

**TITLE (PROVISIONAL)**	Trajectories in long-term conditions accumulation and mortality in older adults: A group-based trajectory modelling approach using the English Longitudinal Study of Ageing
**AUTHORS**	Chalitsios, Christos; Santoso, Cornelia; Nartey, Yvonne; Khan, Nusrat; Simpson, Glenn; Islam, Nazrul; Stuart, Beth; Farmer, Andrew; Dambha-Miller, Hajira

VERSION 1 – REVIEW

**REVIEWER**	Øystein Hetlevik<br>University of Bergen, Department of Global Public Health and Primary Care
**REVIEW RETURNED**	26-Jul-2023

**GENERAL COMMENTS**	The paper "Trajectories of multiple long-term conditions and mortality in older adults: A retrospective cohort study using English Longitudinal Study of Ageing (ELSA)" is based on repeated surveys among older adults (defined as above 50 years), including approx. 15 000 persons. The paper is overall well written and concise about this very important theme.<br>The introduction is brief but to the point introducing MLTC as an increasing challenge and pointing out a lack of longitudinal studies on how patterns of diseases evolve over time. It could have been given some more details about the known risk factors. They also describe the gap in knowledge about trajectories as "critical", which should be justified with more theories about the possible usefulness of identifying such trajectories. What could it add to the known risk factors regarding MLTC in general? The introduction presents aims to (1) classify older adults with MLTC into clusters based on the cumulation of conditions as trajectories over time; (2) characterize clusters, and (3) study associations between derived clusters and all-cause mortality.<br>Concerning the number of participants: Since approx. 12 000 was included from the start, how are the persons included later than 2002 handled? How many persons were participating in the 2004/5 t wave used as the baseline in this study? What about participants included later, and those dying, are they all included in the analyses? And how are the missing answers treated?<br>In the MLTC paragraph (P7L28++)10 conditions are listed and these rather few conditions are mentioned as a limitation earlier, but in line 35++ there is described combination of more specific diseases. Was there a more detailed list of diagnoses available?<br>Covariates (P8L4++) are used from baseline, how is this handled when new persons were included?<br>In the statistical analyses part, the group-based trajectory modeling (GBTM) and fit statistics are described adequately. On page 12 L3 six clusters are mentioned but five were chosen, but figure 2 shows

	only 5 clusters as I can see. Were the fit statistics “worsening“ when adding more clusters? This could have been included in the supplementary table.<br>I understand the model as there is a number between 0 and 10 describing MLTC at each wave of the survey. I miss information detailed information about missing. And also, on how many participants have been followed over how many waves of the survey, see comments above. Some data on this is given at the start of the results, but how many of the persons participated in only one wave? How could they be used in trajectory analyses?<br>Table 1 gives an informative overview of the background variables related to trajectories. It is a bit confusing when the first column gives percentages vertically but the other horizontally. Could this be indicated in some way?<br>The identified clusters were interesting. They seem parallel to each other except for the No-LTC group which has no increase. Aren’t they in sum showing that there is a similar growth in all groups concerning the number of chronic conditions? What does this modeling add to the one-time counting?<br>It is peculiar that there is no group that seems to be one with a stable number for chronic conditions, except for those with no conditions. The discussion should elaborate on these possible problems using these trajectory modeling.<br>On page 14 line 18 it is stated “An interesting finding was that clusters with different initial levels and rates of change in MLTC indicating individual differences in the process of health deterioration.” As mentioned above, doesn’t this show that the numbers are growing with the same slope by time for all, except for those who were “well” when included? This is if I understand this right, not quite what is stated in the conclusion in the abstract: “The development of MLTC and the increase in the number of conditions over time follow distinct trajectories.” It may depend on when you start following the person?<br>There are shown clear associations between cluster and ethnicity, education, and employment, well-known risk factors. The gender difference is more questionable as there is only one significant association between sex and clustering with a CI that is very near to 1, and there is no clear trend. Perhaps this finding is given too much weight?<br>The discussion ends with arguing for this study can help provide knowledge for policy and planning, but one could question the novelty of the result. The topic of MLTC and challenges for health care are surly a very central issue for studying and modeling latent clusters is an interesting method to gain new knowledge, but maybe this way of handling data in the present study did not add much to the well-known risk factors of ethnic minorities and low socioeconomic status?

**REVIEWER**	DAISUKE KATO<br>Mie University Graduate School of Medicine, Department of Family Medicine
**REVIEW RETURNED**	29-Aug-2023

**GENERAL COMMENTS**	I appreciate very much the opportunity to review this paper, which is of great academic value. The authors have, in my opinion, addressed a very important research topic in this study.<br><br>In this study, the authors stated that they have succeeded in identifying an association between multiple long-term conditions (MLTC) trajectories and mortality. I agree with that.

	On the other hand, I would like you to make one change to the description of the paper that I hope will enhance the value of this study.<br><br>In the abstract, the authors stated in the objective part that the aim was to clarify the association between clusters and mortality, but in the conclusion part, they emphasized the importance of identifying older people at high risk of MLTC (i.e., prone to increasing numbers of diseases over time) and providing them with effective interventions.<br><br>In other words, the current description will make it difficult for readers to find consistency in the aims and conclusions of the abstract. I would like the authors to revise this point.<br><br>There were no particular points of concern with regard to the content of the main text.

VERSION 1 – AUTHOR RESPONSE

<u>Reviewer: 1</u>

No	Reviewer comments	Author response
**1**	The paper “Trajectories of multiple long-term conditions and mortality in older adults: A retrospective cohort study using English Longitudinal Study of Ageing (ELSA)” is based on repeated surveys among older adults (defined as above 50 years), including approx. 15 000 persons. The paper is overall well written and concise about this very important theme.	Thank you for the time to review our manuscript.
**2**	The introduction is brief but to the point introducing MLTC as an increasing challenge and pointing out a lack of longitudinal studies on how patterns of diseases evolve over time. It could have been given some more details about the known risk factors. They also describe the gap in knowledge about trajectories as “critical”, which should be justified with more theories about the possible usefulness of identifying such trajectories. The introduction presents aims to (1) classify older adults with MLTC into clusters based on the cumulation of conditions as trajectories over time; (2) characterize clusters, and (3) study associations between derived clusters and all-cause mortality.	Thank you for these insights. We have kept the introduction relatively brief and referenced a range of key sources which shed further light on known risk factors.<br><br>We take onboard the reviewer’s comment about the gap in knowledge being critical. Whilst there is a gap, we acknowledge that a ‘critical gap’ may not reflect the current state of knowledge in this field. We have removed the word ‘critical’ from our text.<br><br>We acknowledge the reviewer’s question about known risk factors regarding MLTC. We think that our study addresses this in both the discussion and conclusion sections of the

		manuscript.
**3**	Concerning the number of participants: Since approx. 12 000 was included from the start, how are the persons included later than 2002 handled? How many persons were participating in the 2004/5 t wave used as the baseline in this study? What about participants included later, and those dying, are they all included in the analyses? And how are the missing answers treated?	Thank you. We mention in the "Data sources and study population" some general information about the ELSA dataset. We mentioned that "it included 12,099 people at study entry in 2002 (wave 1)", so this is not our study baseline population. Below, we mention that our study population was between waves 2 and 6, as wave 2 was the first collecting time point of long-term conditions and the most recent wave with available data on all-cause mortality status.<br><br>In 2004/5 (wave 2=baseline), there were 9,170 participants. We have added this in the results as follows: "*There were 9,170 participants in wave 2...*" (page 8).<br><br>Our study is cross-sectional, so we captured the number of their long-term conditions at a single time point of all participants irrespective of when they were included in wave 2 or later.<br><br>With respect to the missing number of long-term conditions, we excluded these participants from the analyses (n=123). This information has already been included in the manuscript.
**4**	In the MLTC paragraph (P7L28++)10 conditions are listed and these rather few conditions are mentioned as a limitation earlier, but in line 35++ there is described combination of more specific diseases. Was there a more detailed list of diagnoses available?	Thank you for your comment. The 10 LTC we have included in our analysis are the following: hypertension, diabetes, cancer, lung disease, cardiovascular disease, stroke, mental health disorder, arthritis, Parkinson's disease, and dementia.<br><br>However, there were some other LTC including depression, asthma, Alzheimer's disease, heart attack, angina, heart murmur, abnormal heart rhythm, and congestive heart failure, that were combined in the corresponding above 10 categories as the numbers were small, as we have already made this clear in the manuscript.

		No more information is provided in ELSA regarding a more detailed list of diagnoses.
**5**	Covariates (P8L4++) are used from baseline, how is this handled when new persons were included?<br>In the statistical analyses part, the group-based trajectory modeling (GBTM) and fit statistics are described adequately. On page 12 L3 six clusters are mentioned but five were chosen, but figure 2 shows only 5 clusters as I can see. Were the fit statistics "worsening" when adding more clusters? This could have been included in the supplementary table.	Thank you. All the covariates were handled at the baseline for each participant's relevant baseline time point.<br><br>We have added in the supplements the statistics for the sixth cluster which were worse than the fifth cluster, so this is the reason why we chose five clusters.
**6**	I understand the model as there is a number between 0 and 10 describing MLTC at each wave of the survey. I miss information detailed information about missing. And also, on how many participants have been followed over how many waves of the survey, see comments above. Some data on this is given at the start of the results, but how many of the persons participated in only one wave? How could they be used in trajectory analyses?	Thank you. With respect to missing covariates at baseline, we used data provided in the nearest subsequent waves. With respect to the missing number of LTC, we excluded these participants from the analyses (n=123). All this information has already been included in the manuscript.<br><br>We used the participants with participation in at least 2 waves to be able to incorporate into the trajectory model. There were 4,965 participants who participated in all waves and 5,555 who participated in at least 2 waves.
**7**	Table 1 gives an informative overview of the background variables related to trajectories. It is a bit confusing when the first column gives percentages vertically but the other horizontally. Could this be indicated in some way?	Thank you for your comment. We have added the following footnote in Table 1 to make that clearer: "*Note: The percentages in the "total" column are presented vertically, whereas in the other five columns horizontally."*
**8**	The identified clusters were interesting. They seem parallel to each other except for the No-LTC group which has no increase. Aren't they in sum showing that there is a similar growth in all groups concerning the number of chronic conditions? What does this modeling add to the one-time counting?	They might be similar to each other, but the number of long-term conditions they start, and end is different, so this is what our study. The development of multimorbidity and the increase in the number of conditions over time follow distinct trajectories.
**9**	It is peculiar that there is no group that seems to be one with a stable number for chronic conditions, except for those with no conditions. The discussion should elaborate on these possible problems using these trajectory modeling.	Thank you for your comment. There is no group with a stable number of conditions as the study population is older people and it is anticipated that the mean number of conditions will increase as we follow them over time (waves). We have added the following text in the discussion: "*…or due to the older population as it is anticipated that*

		the mean number of conditions will increase as we follow them over time (waves).”
**10**	On page 14 line 18 it is stated “An interesting finding was that clusters with different initial levels and rates of change in MLTC indicating individual differences in the process of health deterioration.” As mentioned above, doesn’t this show that the numbers are growing with the same slope by time for all, except for those who were “well” when included? This is if I understand this right, not quite what is stated in the conclusion in the abstract: “The development of MLTC and the increase in the number of conditions over time follow distinct trajectories.” It may depend on when you start following the person?	Thank you. We have removed the word “increase” as you stated the slopes are similar. However, the development in the mean number of conditions is different.
**11**	There are shown clear associations between cluster and ethnicity, education, and employment, well-known risk factors. The gender difference is more questionable as there is only one significant association between sex and clustering with a CI that is very near to 1, and there is no clear trend. Perhaps this finding is given too much weight?	Thank you for your comment. We have revised the abstract and the key findings in the discussion, not focusing on this specific finding as you suggested.
**12**	The discussion ends with arguing for this study can help provide knowledge for policy and planning, but one could question the novelty of the result. The topic of MLTC and challenges for health care are surly a very central issue for studying and modeling latent clusters is an interesting method to gain new knowledge, but maybe this way of handling data in the present study did not add much to the well-known risk factors of ethnic minorities and low socioeconomic status?	Thank you for this comment. A key aspect we present in our paper, as the reviewer identifies, is the method used in the study to model clusters. In this context, we think that this work does add to the literature on known risk factors although we recognise that this is something that should not be overstated. We have been careful in this paper not to overstate our findings and have situated these in the context of existing literature. In the limitations, we have been careful to acknowledge that ‘the results of this study should be interpreted with some caution,’ although we do feel that our analysis does add to the current evidence base and as such, it is an additional contribution to research and future policy and planning in this field. We have amended the wording in the last paragraph of the manuscript to frame, more precisely, our conclusions: ‘Considering LTC clusters has potential to enable future researchers and practitioners to provide evidence in identifying older adults in England at a higher risk of worsening MLTC over time and further tailoring effective

		interventions for at-risk individuals.’
	**Reviewer: 2**	
**13**	I appreciate very much the opportunity to review this paper, which is of great academic value. The authors have, in my opinion, addressed a very important research topic in this study. In this study, the authors stated that they have succeeded in identifying an association between multiple long-term conditions (MLTC) trajectories and mortality. I agree with that. On the other hand, I would like you to make one change to the description of the paper that I hope will enhance the value of this study. In the abstract, the authors stated in the objective part that the aim was to clarify the association between clusters and mortality, but in the conclusion part, they emphasized the importance of identifying older people at high risk of MLTC (i.e., prone to increasing numbers of diseases over time) and providing them with effective interventions. In other words, the current description will make it difficult for readers to find consistency in the aims and conclusions of the abstract. I would like the authors to revise this point.	Thank you for taking the time to review our paper. The association with mortality is mentioned in the objective part as the 3rd objective. The 1st is to classify older adults into clusters based on accumulating long-term conditions (LTC) as trajectories, and the 2nd to characterise these clusters. Thus, in the conclusion, we state the message of this study regarding the first 2 objectives. We have now added this sentence in terms of the 3rd objective (morality): “*Stratifying risk through clustering will enable practitioners to identify older adults with a higher likelihood of worsening LTC over time to tailor effective interventions to prevent mortality*”.
**14**	There were no particular points of concern with regard to the content of the main text.	Thank you for the time to review our manuscript.

VERSION 2 – REVIEW

**REVIEWER**	Øystein Hetlevik<br>University of Bergen, Department of Global Public Health and Primary Care
**REVIEW RETURNED**	16-Feb-2024

**GENERAL COMMENTS**	In my opinion, the introduction is still short and adding some more information about prior knowledge of the risk factors addressed in this study in the text would be useful.<br>Why is this study now reclassified as a cross-sectional study? Isn't this a longitudinal study, at least concerning trajectories?<br>The paragraph on multimorbidity is still unclear. Is the longer list what the participants were asked about? And then you combine them in the ten groups in this present study? I see that you have a lot of references also here, but this could simply be clarified in the text by rephrasing.<br>Regarding the number of participants, it's still unclear to me how many participants were included from the different waves, and it is mentioned anything about this in the manuscript and only partly in the response letter. Of the trajectories is probably related to how many observations you have for each participant. This information

	should be given in paper. And also that only two waves need to be included. Was no one excluded because they only participated once?<br>Regarding mortality, data seems to be collected from waves 2, 3, 4 and 6 (Why not 5?). I cannot find more information if all these deaths were included in the prediction model. Maybe I am misunderstanding the model, but it seems like the “future” trajectory is used as a predictor. This model should be explained better.<br>Although this modelling is elegant and it's interesting to look at possible latent patterns of disease development, there is still, as mentioned in the first review, a question about the usefulness of this model for practical policy-making or clinical practice. There seems to be a main predictor for the “class- membership”: the number of chronic conditions at baseline. During the study period there is added approximately one condition I all groups (expect one). In the introduction, it is stated that “Understanding the trajectory that an older adult will follow in the progression towards an increased number of LTC could help predict when intervention is needed and inform targeted and earlier preventive interventions.” I'm still not convinced that this model with trajectories could help in this case beyond what's known before based on cross-sectional study of chronic conditions. This is a challenge to the authors.

VERSION 2 – AUTHOR RESPONSE

No	Reviewer comments	Response
1	In my opinion, the introduction is still short and adding some more information about prior knowledge of the risk factors addressed in this study in the text would be useful.	Thank you for this comment. We have added the following text about prior knowledge of known risk factors.<br><br>There are a range of risk factors for multimorbidity, although these may vary ‘quantitively and qualitatively across life stages, ethnicities, sexes, socioeconomic groups and geographies’ (9). The most significant risk factor in multimorbidity, in virtually all contexts, is older age (9,10). Other documented risk factors include low education, obesity, hypertension, depression, and low physical function, which were generally positively associated with multimorbidity (10).
2	Why is this study now reclassified as a cross-sectional study? Isn't this a longitudinal study, at least concerning trajectories?	Thank you for your comment. As per the other reviewer's request, we have changed the title and text to cross-sectional. We have not followed-up the patients but examined the trajectories based on 5 specific time points (Wave 2 to 6). This is a repeated cross-sectional study. If the reviewer would like it changed back to longitudinal, please can the editor kindly liaise with both reviewers to find consensus on how to proceed with these

		differing views on the terminology used.
**3**	The paragraph on multimorbidity is still unclear. Is the longer list what the participants were asked about? And then you combine them in the ten groups in this present study? I see that you have a lot of references also here, but this could simply be clarified in the text by rephrasing.	The following ten conditions were included: hypertension, diabetes, cancer, lung disease, cardiovascular disease, stroke, mental health disorder, arthritis, Parkinson's disease, and dementia. We have added additional clarification on the MLTC paragraph. The references are needed to justify the availability and the selection of the conditions – they also provided much needed additional detail on the MLTC selection.
**4**	Regarding the number of participants, it's still unclear to me how many participants were included from the different waves, and it is mentioned anything about this in the manuscript and only partly in the response letter. Of the trajectories is probably related to how many observations you have for each participant. This information should be given in paper. And also that only two waves need to be included. Was no one excluded because they only participated once?	Thank you. This is an open cohort. The number of participants is different from wave to wave. There were 9,170 participants in wave 2 and we identified 15,091 individuals participating in at least one wave during the follow-up period. The median observation was 4 and everyone included in the model had at least 2 observations.
**5**	Regarding mortality, data seems to be collected from waves 2, 3, 4 and 6 (Why not 5?). I cannot find more information if all these deaths were included in the prediction model. Maybe I am misunderstanding the model, but it seems like the “future” trajectory is used as a predictor. This model should be explained better.	Thank you for this comment. There was no mortality information in the wave 5 in the dataset, hence this is the reason why it has not been included. We have clarified this in the text.<br><br>We state in the manuscript “All-cause mortality was reported by end-of-life interviews on waves 2, 3, 4 and 6 with relatives and friends after death.”<br><br>Then for the identified trajectory clustering we identified the odds of death compared to the relatively healthy cluster.
**6**	Although this modelling is elegant and it's interesting to look at possible latent patterns of disease development, there is still, as mentioned in the first review, a question about the usefulness of this model for practical policy-making or clinical practice. There seems to be a main predictor for the “class- membership”: the number of chronic conditions at baseline. During the study period there is added approximately one condition I all groups (expect one). In the introduction, it is stated that “Understanding the trajectory that an older adult will follow in the	Thank you for this insight. We do not wish to overstate our research findings. In this respect we have removed the sentence: “Understanding the trajectory that an older adult will follow in the progression towards an increased number of LTC could help predict when intervention is needed and inform targeted and earlier preventive interventions.”

	progression towards an increased number of LTC could help predict when intervention is needed and inform targeted and earlier preventive interventions." I'm still not convinced that this model with trajectories could help in this case beyond what's known before based on cross-sectional study of chronic conditions. This is a challenge to the authors.	Additionally, we have modified our conclusions to more precisely contextualise our findings within the current evidence base and in relation to application to policy-making and clinical practice settings:<br><br>'Considering LTC clusters has potential to enable future researchers and practitioners to provide evidence in identifying older adults in England at a higher risk of worsening MLTC over time. Our findings contribute to existing evidence on the need to develop effective tailored interventions for at-risk individuals. Possible responses include targeting ethnic minorities for multimorbidity prevention. Additionally, higher levels of education can also lead to a further decrease in the number of long-term conditions. Policymakers should also commit to increasing MLTC awareness among at-risks groups and care providers.'

VERSION 3 – REVIEW

**REVIEWER**	Øystein Hetlevik<br>University of Bergen, Department of Global Public Health and Primary Care
**REVIEW RETURNED**	04-May-2024

**GENERAL COMMENTS**	The authors have argued well and corrected some points regarding my last review, but still I have a few remarks that probably the editor may conclude without any new review.<br>The first is regarding the design. I am not so concerned about the nomenclature, but really interested to understand what has been done. In my understanding of "trajectory" you have to follow individuals longitudinally (in this case over two or more waves, most of the participants in 4 or more) and data analyses are based on data following these individuals, even if not all are found at all waves. Isn't this a longitudinal study, as the authors wrote from the start ? I just ask, and think authors are the ones that must argue for what is the best description.<br>The next is regarding included participant The authors state: "There were 9,170 participants in wave 2 and we identified 15,091 individuals participating in at least one wave during the follow-up period". As this was an open cohort, there seem to be > 5000 persons that were not part of the baseline (N = 9 170), but in only 129 of the 15091 were not included, no one because they participated only in one wave. Was there no one among that participated in only one wave and not at baseline? What happened to a person who did not participate at baseline and in one wave

	later, is this person not among the 14,962? If so the statement above is not correct. It is probably possible to be more precise here. And still, I do not understand why the authors are not including information about distribution of number of waves for the included participants, as it is mentioned in the response letter at least partly.

VERSION 3 – AUTHOR RESPONSE

No	Reviewer comments	Response
**1**	The authors have argued well and corrected some points regarding my last review, but still I have a few remarks that probably the editor may conclude without any new review.<br><br>The first is regarding the design. I am not so concerned about the nomenclature, but really interested to understand what has been done. In my understanding of "trajectory" you have to follow individuals longitudinally (in this case over two or more waves, most of the participants in 4 or more) and data analyses are based on data following these individuals, even if not all are found at all waves. Isn't this a longitudinal study, as the authors wrote from the start? I just ask, and think authors are the ones that must argue for what is the best description.	Thank you for this comment.<br><br>Thank you for giving us the opportunity to clarify further. Initially, we had specified that this is a longitudinal study; however, after incorporating reviewer's 2 comments we changed that. However, we agree with you, and we have changed all the text accordingly.<br><br>This is in a longitudinal study where we analyse repeatedly collected data from the same population over an extended period of time.
**2**	The next is regarding included participant. The authors state: "There were 9,170 participants in wave 2 and we identified 15,091 individuals participating in at least one wave during the follow-up period". As this was an open cohort, there seem to be > 5000 persons that were not part of the baseline (N = 9 170), but in only 129 of the 15091 were not included, no one because they participated only in one wave. Was there no one among that participated in only one wave and not at baseline? What happened to a person who did not participate at baseline and in one wave later, is this person not among the 14,962? If so, the statement above is not correct. It is probably possible to be more precise here. And still, I do not understand why the authors are not including information about distribution of number of waves for the included participants, as it is mentioned in the response letter at least partly.	Thank you for this comment.<br><br>There were 9,170 participants in wave 2, and we identified 15,091 individuals participating in at least one wave during the follow-up period. Six participants were excluded, as they had no information on LTC. Then, after excluding those (n = 123) with missing data on covariates, 14,962 people were included in the final analysis.<br><br>If a person did not participate at baseline and in one wave later then this person was also included in the analysis.<br><br>Yes, there were some participants not included in baseline but at a wave after

		baseline.<br><br>We have included the following text on the distribution of people in the final analysis by waves:<br><br>“The current analysis included 2688 (18.0%), 529 (3.5%), 4272 (28.5%), 4582 (30.6%) and 2893 (19.3%) people from wave 2, 3 4, 5 and 6, respectively.
